# Proof of Concept for Sustainable Manufacturing of Neural Electrode Array for In Vivo Recording

**DOI:** 10.3390/bios13020280

**Published:** 2023-02-16

**Authors:** Szu-Ying Li, Hsin-Yi Tseng, Bo-Wei Chen, Yu-Chun Lo, Huai-Hsuan Shao, Yen-Ting Wu, Ssu-Ju Li, Ching-Wen Chang, Ta-Chung Liu, Fu-Yu Hsieh, Yi Yang, Yan-Bo Lai, Po-Chun Chen, You-Yin Chen

**Affiliations:** 1 Department of Biomedical Engineering, National Yang Ming Chiao Tung University, No.155, Sec. 2, Linong St., Taipei 112304, Taiwan; 2 The Ph.D. Program in Medical Neuroscience, College of Medical Science and Technology, Taipei Medical University, No. 250 Wu-Xing St., Taipei 11031, Taiwan; 3 Franz Collection Inc., 13F, No. 167, Sec. 5, Ming Sheng E. Rd., Taipei 10589, Taiwan; 4 Department of Biomedical Engineering, Johns Hopkins University, No. 720 Rutland Ave., Baltimore, MD 21205, USA; 5 Department of Materials and Mineral Resources Engineering, National Taipei University of Technology, No. 1, Sec. 3, Zhongxiao E. Rd., Taipei 10608, Taiwan

**Keywords:** fabless manufacturing, laser etching, neural electrode array, carbon footprint

## Abstract

Increasing requirements for neural implantation are helping to expand our understanding of nervous systems and generate new developmental approaches. It is thanks to advanced semiconductor technologies that we can achieve the high-density complementary metal-oxide-semiconductor electrode array for the improvement of the quantity and quality of neural recordings. Although the microfabricated neural implantable device holds much promise in the biosensing field, there are some significant technological challenges. The most advanced neural implantable device relies on complex semiconductor manufacturing processes, which are required for the use of expensive masks and specific clean room facilities. In addition, these processes based on a conventional photolithography technique are suitable for mass production, which is not applicable for custom-made manufacturing in response to individual experimental requirements. The microfabricated complexity of the implantable neural device is increasing, as is the associated energy consumption, and corresponding emissions of carbon dioxide and other greenhouse gases, resulting in environmental deterioration. Herein, we developed a fabless fabricated process for a neural electrode array that was simple, fast, sustainable, and customizable. An effective strategy to produce conductive patterns as the redistribution layers (RDLs) includes implementing microelectrodes, traces, and bonding pads onto the polyimide (PI) substrate by laser micromachining techniques combined with the drop coating of the silver glue to stack the laser grooving lines. The process of electroplating platinum on the RDLs was performed to increase corresponding conductivity. Sequentially, Parylene C was deposited onto the PI substrate to form the insulation layer for the protection of inner RDLs. Following the deposition of Parylene C, the via holes over microelectrodes and the corresponding probe shape of the neural electrode array was also etched by laser micromachining. To increase the neural recording capability, three-dimensional microelectrodes with a high surface area were formed by electroplating gold. Our eco-electrode array showed reliable electrical characteristics of impedance under harsh cyclic bending conditions of over 90 degrees. For in vivo application, our flexible neural electrode array demonstrated more stable and higher neural recording quality and better biocompatibility as well during the 2-week implantation compared with those of the silicon-based neural electrode array. In this study, our proposed eco-manufacturing process for fabricating the neural electrode array reduced 63 times of carbon emissions compared to the traditional semiconductor manufacturing process and provided freedom in the customized design of the implantable electronic devices as well.

## 1. Introduction

Extracellular potential recording and neuromodulation using different stimulation modalities such as electrical, chemical, and optical have a diverse range of uses, ranging from fundamental neuroscience research to neuroengineering for therapeutics [1].

The development of an improved neural implantable device has undergone a tremendous boost through the introduction of silicon-based semiconductor technologies, such as photolithography, thin-film deposition, and blade dicing [2], which have enabled microscale circuit fabrication. Silicon-based neural implantable devices have been used widely in neuroscience research, clinical neurology, and neurosurgery. For neuroscience research, scalability in regard to the length and dimension of neural implantable devices was required owing to differences in species and brain regions [3]. For the clinical investigation of neurological disorders, including Alzheimer’s disease [4], Parkinson’s disease [5], and epilepsy [6,7], specifically designed neural implantable devices such as the stereoelectroencephalography electrode for precise focus localization have emerged [8,9].

Recent years have seen the application of the silicon-based neural implantable device for increasingly versatile functions, such as sensing and actuation using the semiconductor-based micromachining process [10], optoelectronics for optogenetics therapy [11,12], and data communication/transmission during animal interacting behaviors [13]. CMOS applications, for example, combine integrated circuits with recording electrodes to provide more compact input/output connections to the implantable device [10,14,15]. By integrating application-specific integrated circuits or field programmable gate arrays, CMOS implantable devices offer hardware acceleration processing while maintaining their compactness at a highly reasonable energy and space consumption rate [16].

The rapid adoption and widespread use of the Neuropixels device with CMOS manufacturing processing has been applied to research on various species, including mice [17,18,19], rats [17,20,21], and primates [22]. The goal of chronic recordings is to continuously record from the same neurons over the course of days or weeks; however, this has proven challenging for huge populations of neurons. For the enhancement of the quality of neural recordings and the minimization of the breach of the blood–brain barrier and tissue displacement, the fabricated strategies of the neural implantable device were considered through their corresponding dimensions and mechanical properties [23]. The fabrication of tiny neural devices was required for an implantable substrate with a thickness of micron-scale size and good flexibility, which exhibited smaller neural tissue responses around the neural implant [23,24]. Combining the Neuropixels device with a single-shank allowed signals across a plane approximately parallel to the brain surface to be effectively captured by using different geometries, although the ideal approach was sampling the deep brain region in a plane perpendicular to the brain’s surface [25]. To meet this requirement, a Neuropixel 2.0 proved with 5120 recording sites was developed, recording the signal from the same neurons for over two months [26].

However, Neuropixel still depends on the silicon substrate and semiconductor manufacturing process. With higher integration of neural implantable device design and wider application, greater requirements for precise fabrication technology required more expensive manufacturing equipment and increasingly complex designs. The semiconductor manufacturing used a flow production method that was costly, complex, and fixed to extended timelines that could not meet the wide variety of applications with the small-volume and make-to-order manufacturing required for neural implantable devices. Furthermore, the corresponding semiconductor manufacturing involved a complex set of energy-intensive and resource-intensive fabrication processes that generated significant waste and led to a high environmental impact across the full life cycle of implantable devices [27,28]. Although stiff silicon-based neural electrode arrays were widely used in advanced brain science, obvious tissue inflammation was induced by the micromotion at the tissue-electrode contact sites caused by their corresponding mechanical mismatch between the rigid substrate and soft tissue [29]. The flexible neural implants offered hope to reduce progressive inflammation responses for stable neural recordings in the long-term implantation. Many flexible neural implants have been further developed through the incorporation of polymer substrates which have reduced chronic inflammatory tissue reactions. For instance, polyimide (PI) substrates showed promising features of mechanical flexibility, biocompatibility, stability, and dielectric strength [30]. The neural electrode arrays based on PI substrates [31,32,33,34,35,36] provided capabilities that minimized the inflammatory response of mechanically adaptive neural interfaces for stable chronic neural recordings [31,37,38] and made implant shafts stiff enough for surgical insertion [37,39]. Additionally, PI-based neural electrode arrays were particularly amenable to modifications and preparations using existing microfabrication technology due to good electrochemical performance [39,40].

Consequently, three-dimensional (3D)-printed neural implantable devices were designed that could eliminate additional training, the specialized equipment of the fab, and time-consuming microfabrication procedures. It could easily adjust the device geometry or make changes in the electrode arrangement in accordance with experimental needs, reducing the cost and time spent on customization. Yuk, Hyunwoo, et al. developed a soft neural implantable device using materials extruded by 3D printing, consisting of PDMS as the substrate and insulation layer and using PEDOT:PSS ink as the conductive layer with a high-density flexible electronic circuit for in vivo single-unit recordings [41]. Additionally, Lee, Juhyun, et al. reported a 3D printing-based fabrication strategy using a photopolymer that could be modified to target different areas of the brain or scale up for use in larger animal models [3]. Nevertheless, various 3D printing methods have disadvantages in the application of neural implantable devices. For example, the spatter generated by Aerosol Jet Printing would limit the scale of the neural implantable device circuit, and the extrusion 3D printing method was limited by the type and viscosity of the material used. Consequently, a green manufacturing process with customizable neural electrode arrays but with fewer steps and restrictions to the technique was required.

In this study, the neural electrode array was fabricated by a developed eco-friendly manufacturing process by laser micromachining, which reduced the complex steps and requirement of precise machines compared to traditional semiconductor manufacturing processes leading to lower emissions of carbon dioxide. Without the requirement of expensive masks and specific clean room facilities to produce RDLs of a neural electrode array in a micron-dimension, it caused less energy consumption and corresponding emissions of carbon dioxide and other greenhouse gases to achieve an environmentally friendly status. In addition, laser micromachining showed the advantages of simplicity, high efficiency, and low cost, which had a wide spectrum of applications, including green nanoparticle synthesis [42], and the production of a nanostructure surface of biosensor and tissue engineering scaffolds [43,44] in the biomedical field.

Herein, we present the first demonstration of sustainable manufacturing processes for a neural electrode array based on the PI substrate with lithography-free processing for fine conductive RDL traces using laser micromachining and electroplating processes. We successfully poured conductive material into the laser-etched grooves of polymer substrates to form wiring for conductive interconnects and electroplated 3D microelectrodes on the neural electrode array for high-quality neural recordings. Our proposed fabrication provided the advantages of decreased waste production with an eco-friendly and simpler procedure that did not require the restrictive environmental conditions of a cleanroom and enabled rapid design change for the customization of neural electrode arrays than the conventional manufacturing process.

## 2. Materials and Methods

### 2.1. Design and Fabrication of a Neural Electrode Array

**Figure 1:**
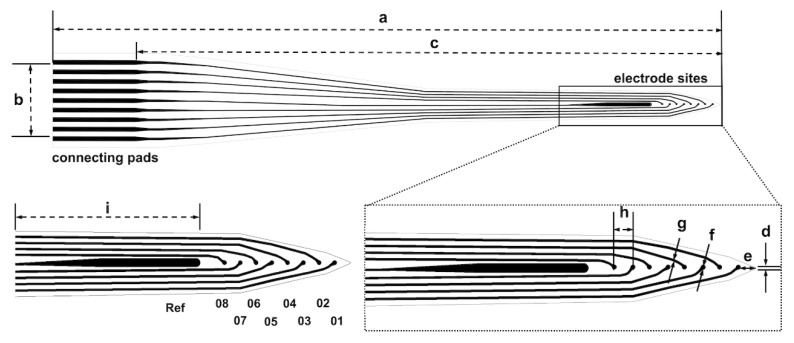
Schematics of the neural electrode array depicted in an AutoCAD layout (not drawn to scale). The neural electrode array was constructed using integrated connecting pads, a long shaft, eight recording sites and a reference electrode; the tip was designed as a 50° tapered angle. Dimensions are listed in Table 1.

Figure 1 shows the schematic view of a neural electrode array as a pattern designed in a computer-aided design file; Table 1 lists the relative specifications. The neural electrode array consisted of eight recording microelectrodes and one reference electrode.

**Figure 2:**
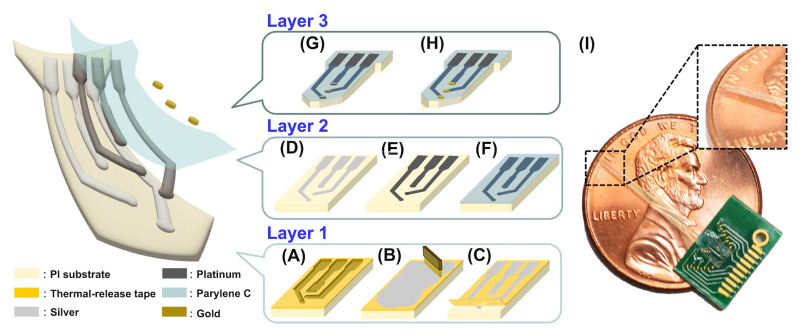
Fabrication flow for the flexible neural electrode array. (**A**) Laser grooving on a PI substrate (light yellow) with a laminated thermal-release tape (dark yellow). (**B**) Drop coating of the silver glue to stack the laser grooved lines, and removal of excess silver glue using a blade. (**C**) Heat release of the protective tape. (**D**) Long-term heating for increasing the electrical conductivity of the silver glue micropatterns (light silver). (**E**) Electroplated platinum depositing on the silver glue-based RDLs (dark silver). (**F**) CVD of Parylene C. (**G**) Laser etching was used to form the via holes over microelectrodes of the neural electrode array and to define the probe-shape. (**H**) Gold deposition onto microelectrodes of the neural electrode array via electroplating (brown). (**I**) The photograph of our fabricated neural electrode array attached to a printed circuit board (PCB) soldering to a small strip connector. The insets show higher magnification images of the tip of the neural electrode array from the black square.

The exploded view of the neural electrode array and the corresponding fabrication scheme with eight steps are shown in Figure 2. A 50-μm thickness polyimide film (PI, CDPI-1530, Chen Da Applied Materials Co., Ltd., Kaohsiung, Taiwan) was selected as the substrate of the neural electrode array. A thermal-release tape (CDPI-1530, Chen Da Applied Materials Co., Ltd., Kaohsiung, Taiwan) with a 25-μm thickness was adhered to the PI substrate as the protective layer to avoid the adhesion of spatters from a melt pool during laser grooving [45]. Then, laser grooving was performed by the 1.5-W ultraviolet (UV) laser (355 nm, 20-ps pulse width, Photonics Industries International Inc., Pittsfield, MA, USA. Laser machine was made by NTS Technology Co., Ltd., Miaoli, Taiwan) at 1 MHz with a beam size of 9 ± 1 µm to produce the 10-μm line grooves in depth for building RDLs of the neural electrode array (Figure 2A). The laser machine with a high-precision motion platform system provided a positioning accuracy of ±1 µm and repeatability of 0.45 µm. Following laser grooving, adhered spatters on the protective tape attached to the PI substrate were removed using sonication in deionized water and 25-W oxygen plasma for 30 sec (PS-3LU, Sheng-Cing Instruments Co., Ltd., Kaohsiung, Taiwan).

For the subsequent formation of wiring conductive interconnects as RDLs (Figure 2B–D), silver glue (GCM-2250EU5, HO MI Specialty Materials Co., Hsinchu, Taiwan) was packed into the grooving lines using drop casting, and the corresponding excess silver glue was removed by a blade (Figure 2B). The PI substrate adhered to the protective tape and was placed in an oven for the heat curing of the silver glue in the line grooves and successive thermal releasing of the protective tape from the PI substrate (Figure 2C). The curing process was first conducted in a vacuum oven at 110 °C and −750 mm Hg for 1 h. The high temperature was for curing the silver glue and vacuum condition to prevent oxidation and to lead the silver glue filling the groove well. To further increase the electrical conductivity of silver glue patterns and prevent oxidation, an additional curing process was conducted in the vacuum oven at 180 °C and −750 mm Hg for 3 h [46,47] (Figure 2D).

Following RDLs’ formation on the PI substrate, platinum electroplating was used to coat the silver glue-based RDLs using an electrochemical analyzer (squidstatTM plus, Admiral Instruments, Tempe, AZ, USA) at room temperature in a conventional three-electrode electrochemical cell containing an array device such as the working electrode, a platinum pad as the counter electrode, and an Ag/AgCl reference electrode. The electroplating procedure was performed in a 5 mm chloroplatinic acid hydrate (PtCl_6_, Merck KGaA, Darmstadt, Germany) and 0.1 M sodium nitrate (NaNO_3_, Merck KGaA, Darmstadt, Germany) solution by applying −1 V potential difference against Ag/AgCl for 60 sec (Figure 2E). Following platinum electroplating, Parylene C (La Chi Enterprise Co., Ltd., New Taipei City, Taiwan) acted as a passivation layer along with an electrical insulation material for our array device [48]. As shown in Figure 2F, a 2-μm thick layer of Parylene C was deposited onto the PI substrate via 3 h of chemical vapor deposition (CVD, LH300, La Chi Enterprise Co., Ltd., New Taipei City, Taiwan) with the usage of 3 g parylene C (C_16_H_14_Cl_2_, La Chi Enterprise Co., Ltd., New Taipei City, Taiwan). The via holes over microelectrodes of the array device and the probe shape of our neural electrode array was defined using laser etching (Figure 2G).

Finally, gold was electroplated onto the platinum-modified silver glue-based RDLs through the via holes to form 3D microelectrodes (Figure 2H). The electrochemical deposition of gold was performed by electroplating at a constant voltage (CV) of 0.65 V for 2, 6, and 10 min using an electrochemical analyzer (squidstat^TM^ plus, Admiral Instruments, Arizona, USA) in 10 mm of tetrachloroauric (III) acid solution (HAuCl_4_, Merck KGaA, Darmstadt, Germany). The CV-electroplating was performed in a typical three-electrode electrochemical cell, comprising an Ag/AgCl reference electrode, platinum pad counter electrode, and neural electrode array as the working electrode. Figure 2I shows that the fabricated flexible neural electrode array was coupled to the PCB.

### 2.2. Characterization of Neural Electrode Array

The electrical properties of the gold-electroplated microelectrodes were determined by measuring impedance at 1 kHz and 20 mV RMS in a phosphate-buffered solution (PBS, Gibco DPBS 1X, Merck KGaA, Darmstadt, Germany) with an electrochemical analyzer (squidstatTM plus, Admiral Instruments, AZ, USA). Observing the impedance at 1 kHz was important for neural recording applications since most electrophysiological signals occur at frequencies of approximately 1 kHz, such as pulses or alternating currents [1]. Scanning electron microscopy (SEM, JSM-7600F, JEOL USA, Inc., Peabody, MA, USA) and atomic force microscopy (AFM, VK-X3000, Keyence Co., Itasca, IL, USA) were, respectively, used to characterize morphology changes and the corresponding surface area of gold-electroplated microelectrodes at different CV electroplating durations.

### 2.3. Bending Test

**Figure 3:**
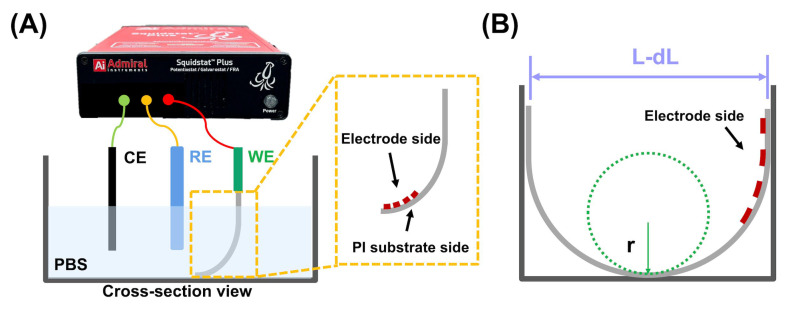
Lab-designed impedance measurement under bending condition. (**A**) Schematic diagram of three-electrode electrochemical cell, consisting of an Ag/AgCl reference electrode, a platinum pad counter electrode, and a bent neural electrode array as the working electrode. The yellow square represents the bending direction to the electrode side of our neural electrode array. (**B**) Defined bending radius, of *r* and length after bending, *L-dL*.

The mechanical flexibility of the gold-electroplated microelectrodes was evaluated using the lab-made bending test (Figure 3A).

The neural electrode array was fixed so that it touched the bottom of a beaker that was filled with PBS to immerse the array device, which was then bent by pushing downward continuously. The resistance change (ΔR) of the frequency at 1 kHz@20 mV rms of the array device was measured with the decreasing bending radius to decide the critical bending radius. The bending radius (r) of the bent shaft of the neural electrode array (Figure 3B) can be approximated using Equation (1) [49].

(1) $$\mathrm{Bending}\;\mathrm{radius}\;\left(r\right)=\frac{L}{2\pi \sqrt{\frac{\mathrm{dL}}{L}-\frac{\pi^{2}h_{s}{}^{2}}{12L^{2}}}}$$

where L, dL/L, and h_s_ denoted the initial length, rate of change in the length, and thickness, respectively, of the shaft of our designed neural electrode array (more detailed specification as described in Table 1).

### 2.4. In Vivo Implantation of the Neural Electrode Array

#### 2.4.1. Animal Preparation and Neural Implantation Surgery (N = 5)

Five male Wister rats were used in this study to examine the neural recording quality and biocompatibility of chronic implants with the lab-designed neural electrode array. All experimental procedures were approved by the Taipei Medical University Institutional Animal Care and Use Committee (IACUC Approval number: LAC-2021-0340) for experimental animals. All animals were 8 weeks old and weighed 250–350 g. The rats were single-housed in a standard plastic rat cage with well-controlled laboratory conditions (12:12 h light/dark cycle with light at 7 a.m.; at a controlled temperature of 20 ± 3 °C) and were fed ad libitum.

For the neural electrode array implantation, rats were anesthetized using an intramuscular injection of 40 mg/kg Zoletil 50 (zolazepam 125 mg/tiletamine 125 mg; virbac, Carros, France) and 8 μg/kg dexdomitor (dexmedetomidine hydrochloride; Pfizer Inc., NY, USA). The rats were placed in a stereotaxic instrument (Model 900, Kopf Instruments, Tujunga, CA, USA). A scalp incision was performed along the midline to expose the bregma and lambda sutures. The PI-based neural electrode array (left hemisphere) and the silicone-based neural electrode array (right hemisphere) [50] were separately implanted into the contralateral ventral posterolateral nucleus (VPL) region (−3 mm anteroposterior, ±3 mm mediolateral, and −6.5 mm dorsoventral), which was used to compare long-term recording quality and biocompatibility between PI- and silicon-based neural electrode arrays. Two implanted neural electrode arrays were anchored to the skull using stainless-steel screws and then secured with dental cement (type 1 class 1; Hygenic Corp., Akron, OH, USA). After a week of recovery, neural recordings were performed on Day 0, Day 3, Day 5, and Day 7 post-one-week recovery, respectively. Finally, the animals were euthanized, and their brain tissues were extracted for immunohistochemistry (IHC) analysis.

#### 2.4.2. Neural Recording and Analysis

To evaluate the quality of neural recording and the biocompatibility of the soft and rigid substrates of the neural electrode arrays in long-term implantation, neuronal extracellular action potentials (spikes) were recorded using our PI-based neural electrode array, and the silicon-based neural electrode array was implanted in the VPL region. Neural signals (spikes) were sampled at 40 kHz, amplified by 10,000–15,000-fold, and band-pass filtered from 250 to 8000 Hz. Neural spikes whose amplitude exceeded a 4-fold standard deviation were determined as neural-discharge signals [51,52]. Then, the amplitudes of these spikes were captured in one window (window = 2 ms), and they were stacked to obtain the spike waveform. The eigenvalues of all the stacked spike waveforms were extracted using the principal component analysis (PCA) method. Feature extraction was performed using the two principal components with the largest eigenvalues. Finally, the K-means clustering method was used to categorize the patterns of the neural spike waveform from different neural cells.

Neural recordings were analyzed offline using MATLAB^®^ software (2020R Mathworks Inc., Natick, MA, USA). Objective measures of signal quality were computed from 30-sec segments of 5 min of continuous recordings using algorithms to estimate the ratio of the background noise level to the signal amplitude (signal-to-noise ratio, SNR). Herein, the background noise was considered as the sum of the electrode noise (electrode–electrolyte interface) [53], instrumentation noise [54], and small amplitude signals from diffuse neural sources (vast distant neurons, synaptic release) [54]. The SNR of the neural signal was defined as the average peak-to-peak amplitude of spikes to the root mean square of the background noise [55,56,57]. According to Chebyshev’s theory, the threshold was set at four standard deviations above or below the mean noise level in this study. That is, in the absence of spike activity, at least 93.75% of the signals would fall within the threshold [55,56].

### 2.5. Immunohistochemistry

The inflammation and survival quantity of neurons was investigated in five rats 14 days after implantation. The rats were perfusion fixed with 4% paraformaldehyde (PFA), anesthetized, and then decapitated. Rat brains were postfixed with 4% PFA in PBS at 4 °C for 24 h. For the dehydration process, the brains were immersed in 20% sucrose solution at 4 °C for 3 days and then sliced into 20-μm thick sections in a direction perpendicular to the long axis of the implanted array device. Tissues were blocked using 0.2% triton X-100 and 10% normal goat sodium in PBS for 30 min. Following this, the tissue was incubated in a primary antibody solution containing the rat anti-glial fibrillary acidic protein (GFAP, Thermo Fisher Scientific, Waltham, MA, USA) 1:500, mouse anti-neuronal nuclear protein (NeuN, Thermo Fisher Scientific, Waltham, MA, USA) 1:250, and rabbit ionized calcium-binding adaptor molecule 1 (Iba1, Life Technologies Co., Eugene, OR, USA) 1:600, overnight in 4 °C. A secondary antibody solution, including 1:500 GFAP Alexa Fluor 488 anti-rat (Thermo Fisher Scientific, Waltham, MA, USA), NeuN, Alexa Fluor 546 anti-mouse (Thermo Fisher Scientific, Waltham, MA, USA), 1:25, Iba1 Alexa Fluor 647 anti-rabbit (Life Technologies Co., Eugene, OR, USA) 1:600, and 4’,6-diamidino-2-phenylindole (DAPI, Life Technologies Co., Eugene, OR, USA) 1:30,000 was used at room temperature for 1 h. After finally being washed with PBS, the samples were mounted on slides.

All images were captured using an automated fluorescence microscope (Olympus BX63, Olympus Co., Tokyo, Japan). To quantify the intensity of the immunomarkers, Image J (https://imagej.nih.gov/ij/, accessed on 2 May 2022) software was used to determine the integral fluorescent intensity around the implantation mark within 100 μm of the brain tissue.

Data were reported as the mean ± standard error of the mean (SEM) obtained from five repeated tests. The histological data were assessed using the Mann–Whitney U-test. The comparison results with *p*-values < 0.05 were statistically significant.

### 2.6. Analysis of Carbon Footprint

The total carbon emissions generated from the developed process in this study and the traditional semiconductor process were calculated via carbon footprint (CFP) tracking for the evaluation of their impact on the environment.

To calculate the total carbon emission, defining the calculation domain excluded the carbon emission generated from the raw material manufacturing, as materials used in the manufacturing process, and waste treatment for each manufacturing stage in the neural electrode array fabrication. In addition, the difference between carbon emissions during transportation and the use of neural electrode arrays fabricated by the two-manufacturing process were minimal and were not included. The carbon footprint of the manufacturing process of the neural electrode array was obtained by collecting the power consumption of the equipment used in each process [58], converting this into carbon emissions using the methodology of electricity as Equation (2), and summing these up as carbon emissions.

(2) $$\mathrm{Input}\;\mathrm{value}\;\left(\frac{\mathrm{kWh}}{\mathrm{Yr}}\right)\times \;F={\mathrm{output}\;\mathrm{value}\;\mathrm{in}\;\mathrm{Kg}\;\mathrm{of}\;\mathrm{CO}}_{2}$$

where the F was emission factor and set as 0.85 for electricity according to ISO 14064 [59].

## 3. Results

### 3.1. Fabless Manufacturing of a Flexible Neural Electrode Array

**Figure 4:**
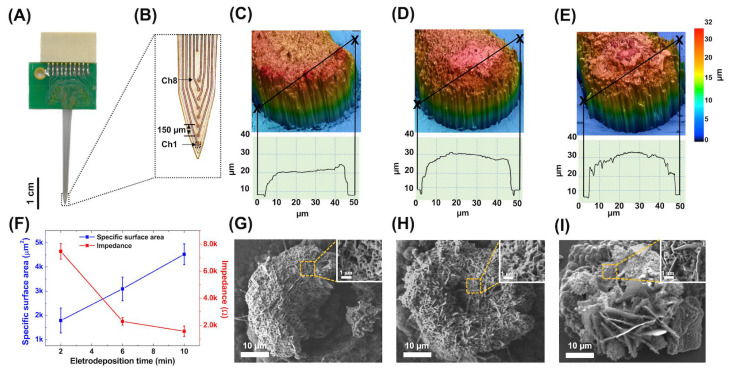
Optical images of the design neural electrode array and impedance increasing using gold electroplating on the electrode site. (**A**) A full view of the neural electrode array. (**B**) The shaft image of neural electrode array in high magnified image. AFM scanning topography and profile of cross-section corresponding to the line in the top image of the different electroplating time of (**C**) 2 min, (**D**) 6 min, and (**E**) 10 min. (**F**) The specific surface area verses the impedance with various electroplating parameters. SEM images of the recording site of the neural electrode array with various electroplating time of gold nanostructures at (**G**) 2 min, (**H**) 6 min, and (**I**) 10 min (Scale bar: 10 µm). The insets showed higher magnification SEM images of gold-nanostructured microelectrode from the yellow square (Scale bar: 1 µm).

The laser grooving was confirmed to have replicated the same pattern as the design drawing (Figure 4A). The elongated shaft allowed the neural electrode array to be implanted, and eight microelectrodes were used to record electrophysiology signals, as shown in Figure 4B.

The specific surface area of electrode sites was specifically increased to enhance the sensitivity of neural signal recording; therefore, the gold nanostructure was deposited on the electrode by electroplating to increase the surface area of microelectrodes in the array device. AFM imaging (Figure 4C–E) showed that with the increasing electroplating time from 2 min to 6 and 10 min, the microelectrode height increased from 17.94 μm to 22.27 and 26.88 μm, respectively. In addition, an increasing surface roughness from 0.416 to 0.917 and 1.093 μm was observed for the electroplating time, respectively.

The corresponding changes in specific surface areas and impedance with the increase in electroplating duration are shown in Figure 4F. In addition, by comparing the resistance under different electroplating times at 1 kHz, the specific surface area of the electrode was inversely proportional to its resistance. A stepped-up increase in the surface area with a slowly decreasing impedance was found after the 6 min deposition time. SEM imaging (Figure 4G–I) showed a growing number of gold nanostructures deposited on the electrode with the increase in the electroplating time from 2 to 10 min with excessive gold deposition outside of the electrode site area (Figure 4I); this might cause a short circuit that fails to record electrophysiology signals. The optimum gold electroplating time of 6 min was used for further testing and animal experiments.

### 3.2. Bending Challenge

**Figure 5:**
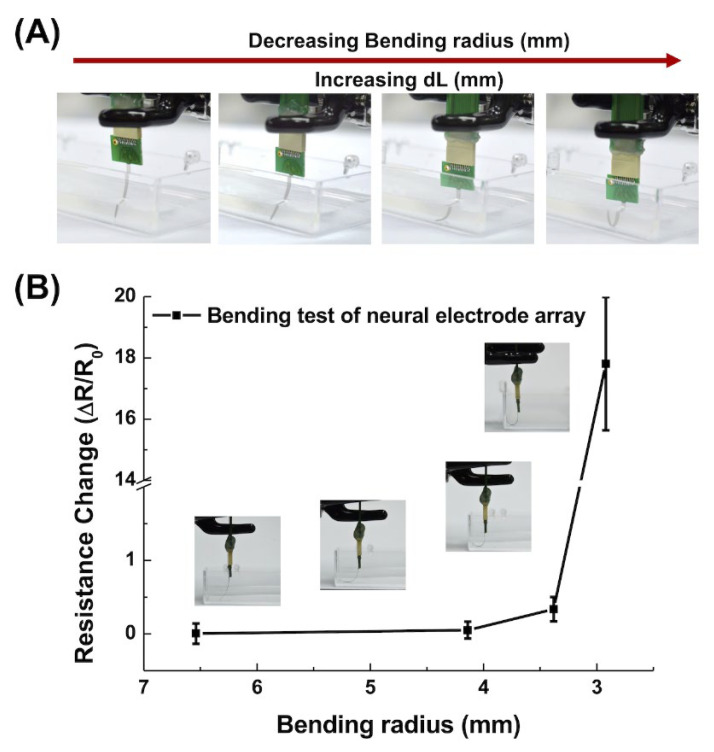
Bending test results with a decreasing bending radius for the neural electrode array. (**A**) Schematic illustration of the structural features of the flexible neural electrode array during a wide range of bending. (**B**) Rate of change in the resistance (∆R/R_0_) of the neural electrode array at various bending radius.

The shaft of the neural electrode array was bent by extrusion from the bottom of the beaker (Figure 5A). The designed neural electrode array could return to the initial state after a large bending radius, demonstrating its flexibility. The resistance change (∆R/R0) was within 0.1–1 for a bending radius from 6.54 to 3.38 mm, as shown in Figure 5B, which showed corresponding optical images of the bending situation. The resistance remained constant for a wide range of bending radii, and no cracking was generated in the shaft of the neural electrode array. However, a dramatic increase in resistance was observed at 17.62 when the bending radius was <3 mm, which was caused by the cracking of the silver wiring conductive interconnects of the RDLs.

These results demonstrated the bendability of the neural electrode array, indicating the applicability of implantation. This characteristic could be attributed to the flexibility of the PI substrate and the compatibility between the conductive layer and the substrate.

### 3.3. In Vivo Electrophysiological Signal Recording

**Figure 6:**
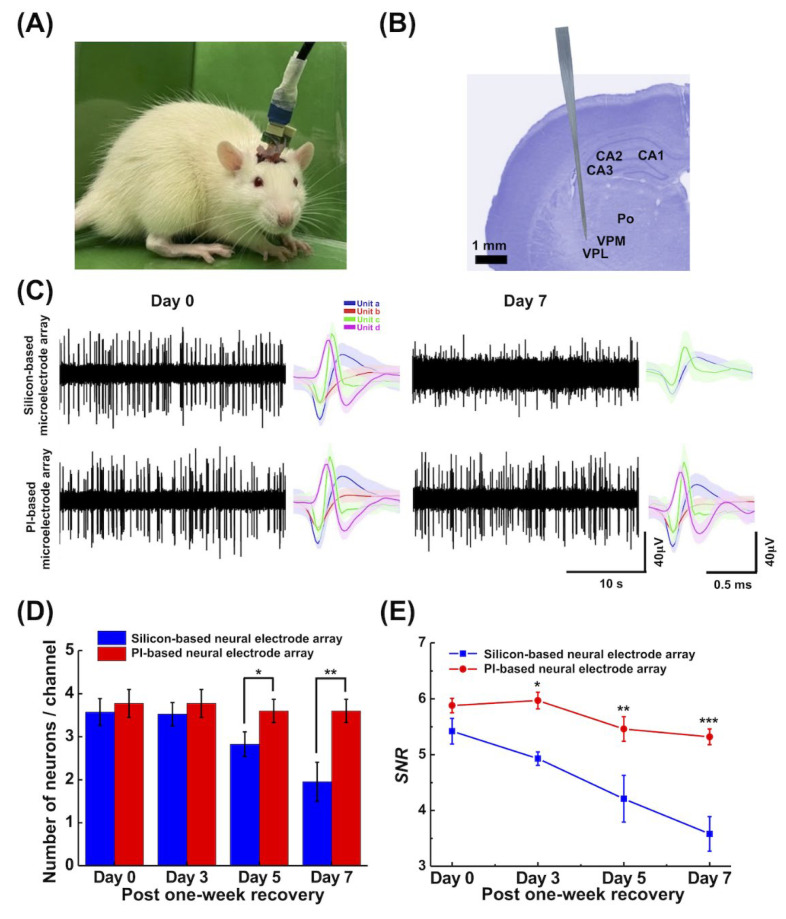
In vivo neural recordings and evaluation of recording quality. (**A**) A freely moving rat with implanted neural electrode arrays. (**B**) Photomicrograph of in situ location of the neural electrode array in the Nissl-stained section at 3 mm posterior to bregma. (**C**) Representative example of 30-sec neural spike trains and their corresponding spike waveforms from sorted neurons at Day 0 and Day 7 post-recovery, which showed no apparent decrease in the PI-based neural electrode array but exhibited a significant difference in the silicon-based neural electrode array (**D**) Number of recorded neurons from silicon-based and PI-based neural electrode arrays within 7 days after a week recovery. (* *p* < 0.05, ** *p* < 0.01) (**E**) SNRs of silicon- and PI-based neural electrode arrays at Day 0, Day 3, Day 5, and Day 7 post-recovery. (* *p* < 0.05, ** *p* < 0.01, *** *p* < 0.001). Abbreviations: Po, posterior thalamus nuclear group; VPM, ventral posteromedial thalamic nucleus; VPL, ventral posterolateral thalamic nucleus.

Neural recordings were performed in awake animals (Figure 6A)**.** For the comparison of neural recording quality between the PI- and silicon-based neural electrode arrays, the animals received neural implantation in bilateral thalamic VPL nuclei (Figure 6B) with neural recording for 7 days after a one-week recovery. Here, Figure 6C shows the representative spontaneous spike firing recorded by PI- and silicon-based neural electrode arrays at Day 0 and Day 7 post-one-week recovery. Following 7 days of recording, the recording quality exhibited increasing background noise, and a fewer number of neurons were recorded by the silicon-based neural electrode array, indicating that the recording quality of this array device was deceased within 7 days after a week’s recovery. However, the PI-based neural electrode array maintained a stable recording quality.

The mean number of sorted neurons at Day 0, Day 3, Day 5, and Day 7 post one week of recovery, recorded by both the PI- and silicon-based neural electrode arrays, are shown in Figure 6D. Significantly fewer neurons were recorded with the silicon-based neural electrode arrays that were found compared to those of the PI-based neural electrode array on Day 5 and Day 7 post at one-week recovery. The SNRs of the silicon-based and PI-based neural electrode arrays were measured on Day 0, Day 3, Day 5, and Day 7 post-one-week recovery (Figure 6E). We found stable and high SNRs of the recorded neural signal with the PI-based neural electrode array during a 7-day recording duration. However, SNRs continued to decrease over time following neural recordings with the silicon-based neural electrode.

### 3.4. Immunohistochemistry

**Figure 7:**
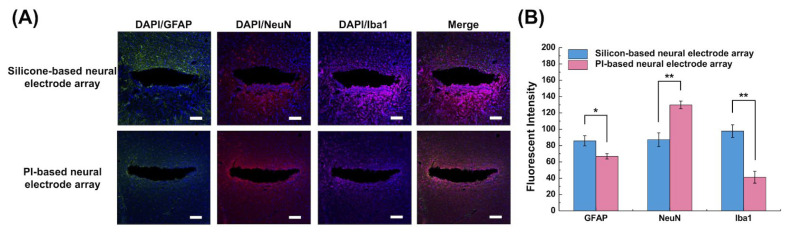
Biocompatibility comparison between silicon-based and PI-based neural electrode arrays. (**A**) Representative images of GFAP, NeuN, and Iba1 with DAPI around the neural electrode array region after 14 days of implantation (scale bar: 100 μm). (**B**) The integral of fluorescent intensity within 100 μm of the implanted region for GFAP, NeuN, and Iba1 staining. (* *p* < 0.05, ** *p* < 0.01).

The pathological changes in our PI-based neural electrode array were compared with those of the silicon-based neural electrode array 14 days after the implantation using immunostaining. The biological compatibilities of our flexible neural electrode array were verified by evaluating GFAP, NeuN, and Iba1 staining (representing activated astrocytes, neurons, and microglia, respectively, and by fluorescence imaging and fluorescence quantification. Based on the GFAP and Iba1 staining, more astrocytes and a large number of activated inflammatory factors were found around the periphery of the wound with the silicon-based neural electrode array (Figure 7A) than were seen around the scars of the PI-based neural electrode array. A significantly higher fluorescence intensity of GFAP and Iba1 was present with the silicon-based neural electrode array than those in the PI-based neural electrode arrays in the fluorescence quantitative results from a 100-μm periphery of the wound (Figure 7B).

A more intense inflammatory reaction was caused by the rigid substrate of the silicon-based neural electrode array, whereas the soft substrate of the PI-based neural electrode array caused less mechanical damage to the tissue during implantation and micromotion within 14 days of implantation. Notably, the quantitative fluorescence result of NeuN for the PI-based neural electrode array was significantly higher than that of the silicon-based group after 14 days of implantation, indicating that the number of nerves that survived the post-implantation of the PI-based neural electrode array was considerably greater than that of the silicon-based neural electrode array. This verified that the PI-based neural electrode array was suitable for implantation and was biocompatible.

### 3.5. Carbon Footprint

The carbon dioxide emissions from the semiconductor and the proposed fabless manufacturing processes of the neural electrode array are shown in Table 2; the calculated power consumption per hour for the fab was 4.7-fold more than the total power consumption of the fabless neural electrode array manufacturing process in this paper. Therefore, the total carbon dioxide emitted from the semiconductor manufacturing process was approximately 15.39 kg, of which 38.7% was emitted from the wafer fabrication. However, as the carbon footprint calculation excluded the carbon dioxide emission from the raw materials and as its manufacture produced carbon emissions, wastewater, and garbage from the raw materials at each processing stage, the true carbon dioxide emissions were probably much higher than 15.39 kg.

The fabless manufacturing process for a neural electrode array in this study emitted 0.2434 kg of carbon dioxide. Additionally, the CVD process consumed the maximum power because of the need to equip the system with a vacuum device and a high-power device to form a vapor phase of plating, followed by sintering the conductive material, which took 3 h at 180 °C. However, when compared with the semiconductor manufacturing process, the new neural electrode array manufacturing process reduced the carbon emission by approximately 63-fold, proving that the fabless manufacturing process described herein is eco-friendly.

## 4. Discussion

### 4.1. Electrochemically Modified Electrodes with Gold Nanostructure and Influence for the Surface Area

Gold is a material with high electrical conductivity, chemical and thermal stability, and biocompatibility [61] and is consequently widely used in specific electronic, optical, thermal, catalytic, and magnetic functions [62,63]. A variety of techniques, such as direct electrostatic assembly, covalent linkage, polymer entrapment or comixing, sol-gel, and electroplating, could produce modified electrodes with the gold nanostructure. Electroplating was one of the most popular techniques owing to the ease of use and variety of nanostructures of gold deposition obtained using different voltages or plating techniques [64,65]. Meanwhile, adjustable plating duration facilitated the control of the deposited gold thickness on the microelectrode of our flexible neural electrode array.

A lower electrode impedance on a neural implantable device was preferable for recording neural signals since this increased the sensitivity of detection for low-level neural signals [66,67]. The impedance was reciprocally related to the surface area of the electrode, and a lowered impedance could thus be achieved by increasing the surface area of electrodes on neural implantable devices. Although approaches to reducing electrode impedance without increasing the size of the electrode have been developed, the planar size of the electrodes should still be kept to a minimum to restrict the size of neural implantable devices and avoid tissue injury. In order to decrease electrode impedance, highly conductive materials have been deposited on electrodes [68,69] to form micro- or nanotopography by roughening the electrode surface [70,71] for use in neural recording and stimulation both in vivo and in vitro [72,73,74]. Herein, the signal recording site on the neural electrode array was electrochemically modified: under CV electroplating, the deposition of gold nanostructures increased with the increasing electroplating duration. A great number of gold nanoparticles were deposited on the electrode to form a porous and 3D nanostructure to increase the roughness and elevate the total electrode area on the original electrode [75], which subsequently increased the specific surface area of the electrode and decreased the impedance [76]. However, 10 min electroplating produced a slowly decreasing impedance and irregular depositing of the gold nanostructure deposited at the electrode site, which might cause instability in the structure. In addition, the over-deposition of the gold nanostructure out of the electrode could increase the possibility of a circuit short.

### 4.2. Electric Performance of the Neural Electrode Array under Bending Condition

The stability of the neural electrode array while in use was verified via a bending test. The soft PI substrate enabled the neural electrode array to be substantially bent. Continuous bending altered the length of the neural electrode array, and the bending radius decreased accordingly with a drastic change in impedance because the resistance of the metal layer to mechanical stress was less than that of the polymer substrate [77]. Thus, the metal layer assembled on a soft polymer substrate undergoing the same deformation challenge would be prone to irreversible and catastrophic changes, such as fracture, delamination, or sliding [78]. Inevitably, in order to reduce the damage to neurons caused by neural electrode arrays during implantation, the demand for soft substrates has increased, and most of the neural electrode arrays were composed of soft polymer substrates with high-conductivity metal designed to be used with a 90° bend [79]. However, the neural electrode array in this study achieved a wide range of bending radius without affecting the electrical properties even if the bending angle was over 90° Our neural electrode array could not only achieve large-angle bending but also maintain the electrical properties after bending because of the 3D space (known as RDLs) of the subsequent metal layer produced on the substrate by laser grooving. This ensured that the metal layer was covered on three sides for circuit protection and that only the upward side was exposed. The increase in the contact area of the two various material layers avoided the sliding and deformation of the metal layer during bending. Compared with the traditional 3D printing technology that also utilized a fabless process, this method directly printed the conductive material onto the substrate to form a metal layer with only one contact side between the metal layer and the substrate, which increased the possibility of the sliding and deformation of the metal layer after bending the neural electrode array. Thus, the neural electrode array that was fabricated in this study was shown to remain stable while bending. In addition, Parylene C evaporated onto the metal layer and not only served as insulation owing to the high electrical resistivity but also protected the metal layer because of the high degree of mechanical flexibility [80].

### 4.3. Long-Term Implantation with Stable Neural Recording and Biocompatibility

To demonstrate the difference in the use and biocompatibility of the neural electrode array produced by the soft and rigid substrate, we simultaneously implanted PI- and silicon-based neural electrode arrays in the left and right thalamic VPL nuclei in the rats to compare the recording of neural signals. The animals were euthanized 14 days after the implantation to analyze the safety of the two substrates.

In the results, it was found that the rigid substrates of the silicon-based neural electrode array might affect the stability of recorded neural signals during long-term neural recordings. Previous studies suggested that the inflammatory reaction at the implantation region was mainly caused by the micromotion between the tissue and the electrode interface, which might cause serious damage to the brain tissue, and it was also closely related to the long-term recording of neural signals, which used the neural electrode arrays in previous studies since rigid substrates were used in most of the microfabrication techniques [81]. Thus, the generation of inflammation affected the quality of neural signals [82] that were assessed by immunohistochemistry results. A significant mechanical mismatch existed between the rigidity of silicon-based or metal neural implantable devices and that of the soft cortical tissue that could cause continuous harm to the extracellular matrix, neurons, and microvasculature of the brain [83,84]. The subsequent generation of glial cell encapsulation and glial scars surrounding the electrode and the retraction of neurons from it hindered high-quality recording [85].

In addition, another source of failure in long-term neural recordings was the oxidation or functional degradation of materials, such as the oxidation of the exposed metal layer and the attachment of proteins to the electrodes. Hence, to avoid early failure, the contact between the metal layer and the tissue was protected by using a coating that acted as an insulating layer. Parylene C has been used as a coating layer for long-term implants for six months [86] and was biocompatible [87,88]. Therefore, in addition to utilizing the highly biocompatible PI soft substrate, Parylene C was evaporated onto the neural electrode array. Compared with the silicon-based neural electrode array, our PI-based neural electrode array with an insulative and protective layer of Parylene C significantly reduced the degree of inflammation and maintained stability in long-term neural recordings, in which the critical factors for the chronic presentation of an implant could induce an immune response to a persistent foreign object in the brain [89,90].

### 4.4. Evaluation the Carbon Footprint of Manufacturing Process of for Neural Electrode Array

The seriousness of the greenhouse effect has made the discussion and reduction in carbon footprints a global focus [91]. The neural electrode array fabricated by the semiconductor manufacturing process should be completed in the fab, which required strict environmental maintenance, including air cooling, airflow purging, water cooling, and an ultra-pure water purification system, which all have carbon footprints that could not be eliminated [60]. In addition, the high carbon emissions of the semiconductor manufacturing process were attributed to the complex procedures used in the process, including cleaning, oxidation, photolithography, etching, and thin film deposition, which involved the simultaneous use of multiple high-powered precision equipment. The wafer manufacturing process usually involves the use and emission of perfluorocarbons with high global warming potential. Thus, semiconductor fabrication was an environmentally detrimental process with high carbon emissions.

In the manufacturing process developed in this study, moving the manufacturing environment out of the fab shortened the processing time and simplified the process steps, and simultaneously used less precision equipment while maintaining the ability to fabricate micron-scale electrodes of our neural electrode array. Thus, our study not only presented the concept of a green manufacturing process but also reduced the carbon emissions produced by the fabrication of a neural electrode array compared with the current semiconductor manufacturing process.

## 5. Conclusions

In this study, we built a manufacturing process for a rapid, fable, and eco-friendly fabrication of neural electrode arrays. The current semiconductor manufacturing process involves toxic chemicals and complex steps with the requirement of precision equipment, which results in high carbon emissions. Herein, we reduced the steps of the fabricated process, the usage of chemicals and equipment, and the 63-fold carbon emission than the standard semiconductor manufacturing process, which proved the concept of sustainability and an environmentally friendly approach. In addition, our manufacturing process showed the impact on consumer neuroscience research. To overcome the inability of the make-to-order semiconductor process, we successfully obtained the implantable device without the use of expensive masks and specific clean room facilities and met the customization requirement. Therefore, the ability to produce a fast production of PI-based neural electrode array without a strict environment control fab accelerated the expansion of neural science. Herein, the designed PI-based neural electrode array demonstrated the more stable quality of neural recordings and better biocompatibility for chronic implantation compared with those of the silicone-based neural electrode array, leading to numerous attention and recognition across the mainstream academic community.

## Figures and Tables

**Table 1:** Specifications of the neural electrode array.

Dimension	Value
Number of sites	9
Full length (a) (mm)	14
Width of bonding area (b) (mm)	1.97
Shaft length (c) (mm)	12.8
Tip width (d) (μm)	10
Distance (e) (μm)	150
microelectrode diameter (f) (μm)	40
Width of interconnects of RDL (g) (μm)	20
Microelectrode spacing (h) (μm)	150
Area of reference electrode (i) (μm^2^)	115,350

**Table 2:** Carbon footprint at each stage of neural electrode array fabrication with the semiconductor commercial manufacturing process and fabless manufacturing process in this study.

	Steps	Electric Power Consumption (Whr)	Carbon Footprint (Kg CO_2_/pcs Neural Electrode Array)
Semiconductor manufacturing process [60]	Wafer fabrication	504,960/wafer	4.292
	Semiconductor fabrication	1,305,556/wafer	11.097
	Fab	135,040	1.148
Fabless manufacturing process (this work)	Laser etching fabricated RDLs	12.450	1.058 × 10^−4^
	Cleaning using O_2_ plasma	0.208	1.764 × 10^−6^
	Sintering in an oven	2200	1.900 × 10^−2^
	Parylene C deposition by CVD	26,400	2.240 × 10^−1^
	CV electroplating platinum	2.939	2.498 × 10^−5^
	CV electroplating gold	17.600	1.496 × 10^−4^

## Data Availability

The datasets generated in this study are available from the corresponding author on reasonable request.
